# Systematic review of the economic evaluation model of assisted reproductive technology

**DOI:** 10.1186/s13561-024-00509-3

**Published:** 2024-05-20

**Authors:** Yuxin Si, Tao Tan, Kexue Pu

**Affiliations:** 1 School of Medical Informatics, Chongqing Medical University, Chongqing, 400016 China; 2 Chongqing Health Statistics Information Center, Chongqing, 401120 China

**Keywords:** Assisted reproductive technology, Economic evaluation, Model methodology, Systematic review

## Abstract

**Background:**

With the increasing demand for fertility services, it is urgent to select the most cost-effective assisted reproductive technology (ART) treatment plan and include it in medical insurance. Economic evaluation reports are an important reference for medical insurance negotiation. The aim of this study is to systematically evaluate the economic evaluation research of ART, analyze the existing shortcomings, and provide a reference for the economic evaluation of ART.

**Methods:**

PubMed, EMbase, Web of Science, Cochrane Library and ScienceDirect databases were searched for relevant articles on the economic evaluation of ART. These articles were screened, and their quality was evaluated based on the Comprehensive Health Economics Evaluation Report Standard (CHEERS 2022), and the data on the basic characteristics, model characteristics and other aspects of the included studies were summarized.

**Results:**

One hundred and two related articles were obtained in the preliminary search, but based on the inclusion criteria, 12 studies were used for the analysis, of which nine used the decision tree model. The model parameters were mainly derived from published literature and included retrospective clinical data of patients. Only two studies included direct non-medical and indirect costs in the cost measurement. Live birth rate was used as an outcome indicator in half of the studies.

**Conclusion:**

Suggesting the setting of the threshold range in the field of fertility should be actively discussed, and the monetary value of each live birth is assumed to be in a certain range when the WTP threshold for fertility is uncertain. The range of the parameter sources should be expanded. Direct non-medical and indirect costs should be included in the calculation of costs, and the analysis should be carried out from the perspective of the whole society. In the evaluation of clinical effect, the effectiveness and safety indexes should be selected for a comprehensive evaluation, thereby making the evaluation more comprehensive and reliable. At least subgroup analysis based on age stratification should be considered in the relevant economic evaluation.

## Introduction

Population development is about the rise and decline of the well-being of individuals and an entire nation. China is the world’s largest population in human history, throughout its tremendous achievement in economic and social development, population factors play an important role [1]. According to the data released by the National Bureau of Statistics in 2022, 10.62 million people were born in 2021, with a birth rate of 7.52%, hitting a 60-year low. The natural population growth rate dropped to 0.34%, and the seventh national census in 2020 revealed that the proportion of the aging population aged 65 and above had risen to 13.5% [2]. At the end of 2021, the proportion of the population aged 65 and above in China increased to 14.2% [3], which means that the trend of the aging population continues to deepen and China has officially entered the aging society, which will bring many social problems, such as pension problems, housing problems, and labor shortage, thereby affecting the sustainable development of China’s economy. To actively cope with an aging population, China introduced a series of major policy initiatives, from the “two-child policy” and “selective two-child policy” to the “universal two-child policy” and then a “three-child policy” this year. China’s birth policy is gradually relaxing to encourage birth, but the effect is not significant largely because of the following reasons: “can’t give birth,” “dare not give birth,” and “can’t afford to give birth.”

On a global scale, the world fertility situation is facing multiple trends and challenges. The United Nations Population Fund (UNFPA) reports that 2/3 of the world’s people live in countries with low fertility rates, and many developed countries and regions are facing declining fertility rates. Japan, Italy, Germany and other countries continue to have low fertility rates, which is known as the phenomenon of “fewer children”. This is partly due to factors such as economic development, higher levels of education, increased participation of women in the profession and the rising cost of living. Although fertility rates are relatively high in many developing countries, fertility patterns are also changing, with family sizes tending to shrink and the desire to have children declining. The countries with the highest fertility rates are all in Africa, with Niger (6.7) having the highest fertility rate [4].

According to information released by the World Health Organization (WHO), about 17.8% of adults in high-income countries suffer from infertility, and the rate is slightly lower at 16.5% in low - and middle-income countries. The incidence of infertility in China is 7–10% in 2021. WHO has predicted that in the 21st century, infertility will become the third most serious disease after tumor and cardiovascular disease [5]. Since the first successful use of in vitro fertilization (IVF) in 1978, assisted reproductive technology (ART) has become an important part of modern medicine, playing an important role in family planning [6]. In 2017, the International Committee Monitoring ARTs (ICMART) issued a new and clear definition of ART as the processing of human oocytes and spermatozoa or embryos in vitro for reproduction purposes. This includes, but is not limited to, IVF-embryo transfer (IVF-ET); intracytoplasmic sperm injection (ICSI); embryo biopsy; preimplantation genetic testing (PGT); assisted incubation; gamete intrafallopian transfer (GIFT); intratubal transfer of fertilized eggs; cryopreservation of gametes and embryos, semen, and oocyte; embryo donation; and surrogacy [7]. However, many families are not interested in ART because of its multiple and expensive costs, long cycle time, and uncertain treatment results [8]. In February 2022, Beijing took the lead in including 16 ART items into medical insurance [9], which is of great help to the popularization and promotion of ART and reduces the treatment cost of infertility within a certain range. However, it has not been included in medical insurance in most developing countries and regions, so the cost problem of ART still needs to be solved.

With the constant improvement of fertility requirements, selecting the most cost-effective of ART therapy and including it in health care is very urgent. In recent years, scholars at home and abroad have conducted an economic evaluation of ART at different levels, aiming to find the treatment measures with economic benefits to make reference for clinical decision-making and reduce the burden on society, medical institutions, and patients. These economic evaluation reports will become an important reference basis for medical insurance negotiations. However, the quality of these economic evaluation reports determines the magnitude of their reference value. By systematically reviewing the economic research on ART at home and abroad, it is helpful to provide reference for future relevant economic research. At present, there is no a systematic evaluation of the economic evaluation of ART at home and abroad.

## Objectives

This systematic review aims to summarize the current research on the economic evaluation of ART at home and abroad by identifying, evaluating, and synthesizing data from ART-related economic studies. It then analyzes the existing shortcomings and provides a basis and reference for the economic evaluation of different treatment measures of ART.

## Methods

### Study design

The preferred reporting items for systematic reviews and meta-analysis [10] are used as the basis for reporting the review. We searched the relevant articles and repeatedly read the articles included in the analysis. Then, we extracted, integrated, and summarized the valuable information, ultimately drawing impactful and persuasive conclusions.

### Literature retrieval strategy

**Fig. 1:**
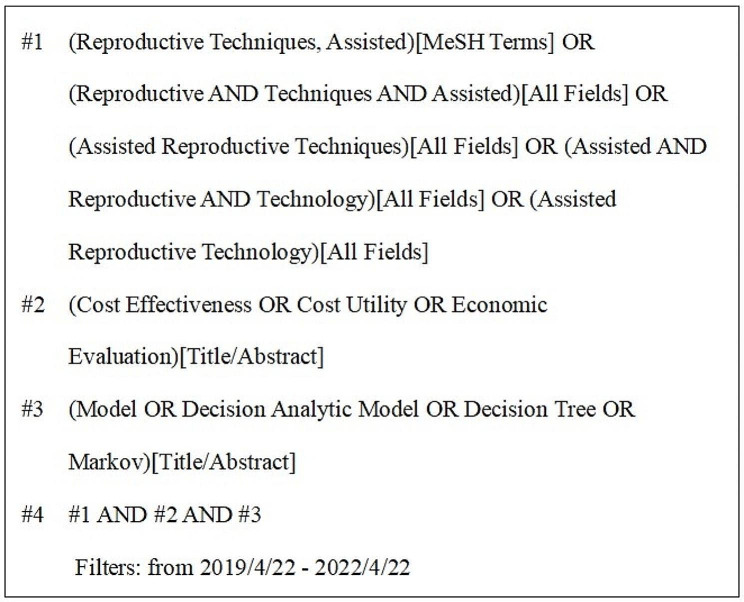
Search strategy in PubMed

PubMed, EMbase, Web of Science, Cochrane Library, and ScienceDirect databases were searched for economic reviews of ART. The search terms include ART, cost-effectiveness, cost-utility, economic evaluation, model, decision analytic model, decision tree, and Markov. The terms were searched in different combinations. To ensure the timeliness and advance of the articles, the search time was limited to three years, from April 22, 2019 to April 22, 2022. Using PubMed as an example, the specific retrieval strategy is depicted in Fig. 1.

### Inclusion criteria

(1) Population (P): low-fertility patients receiving ART.

(2) Intervention (I) and Comparison (C): the existing ART treatment programs.

(3) Outcome (O): pregnancy rate, live birth rate, cumulative live birth rate and other birth outcome indicators.

(4) Study design (S): the model-based health economics evaluation of ART.

### Exclusion criteria

The exclusion criteria are as follows: (1) non-English literature; (2) case reports, conferences, lectures, reviews, comments, etc.; (3) research on unused modeling techniques and (4) the full text cannot be obtained.

### Literature screening and data extraction

Two evaluators independently screened the literature, extracted the data, cross-checked, and asked for the opinions of a third party to help solve any disagreement. Articles from various sources were summarized, and duplicates were eliminated. First, the title and abstract of articles were read to exclude obviously irrelevant articles, and then, the full text was further read to determine whether the articles were included in the analysis based on the inclusion and exclusion criteria. The included studies were summarized and analyzed qualitatively. The bases of the data extraction mainly include the following. (1) basis features of the included studies are first author, country, publication year, research perspective, target population, and comparison scheme of ART. (2) The model characteristics of the included studies are model type, health status included in the model structure, cycle period, time horizon, discount rate, and willingness to pay (WTP) value. (3) The model parameters of the included studies are data sources of cost/effect/state transition probability and cost inclusion items. (4) The results of the included studies are the outcome indicators, cost–benefit analysis, and sensitivity analysis.

### Quality of included studies

Two scholars evaluated the quality of the included articles. The evaluation was carried out based on the Consolidated Health Economic Evaluation Reporting Standards 2022 (CHEERS 2022) [11]. The list contains 28 items divided into seven main categories—title, abstract, introduction, methods, results, discussion, and other relevant information, such as the source of funding and conflicts of interest [11]. Based on the items listed, we determine whether there are chapters in the article where relevant information can be found. If they can be found, it meets the standard requirements and is marked as “Yes” and otherwise “No.” The higher the proportion of “Yes,” the higher the quality of the article [11, 12].

## Results

**Fig. 2:**
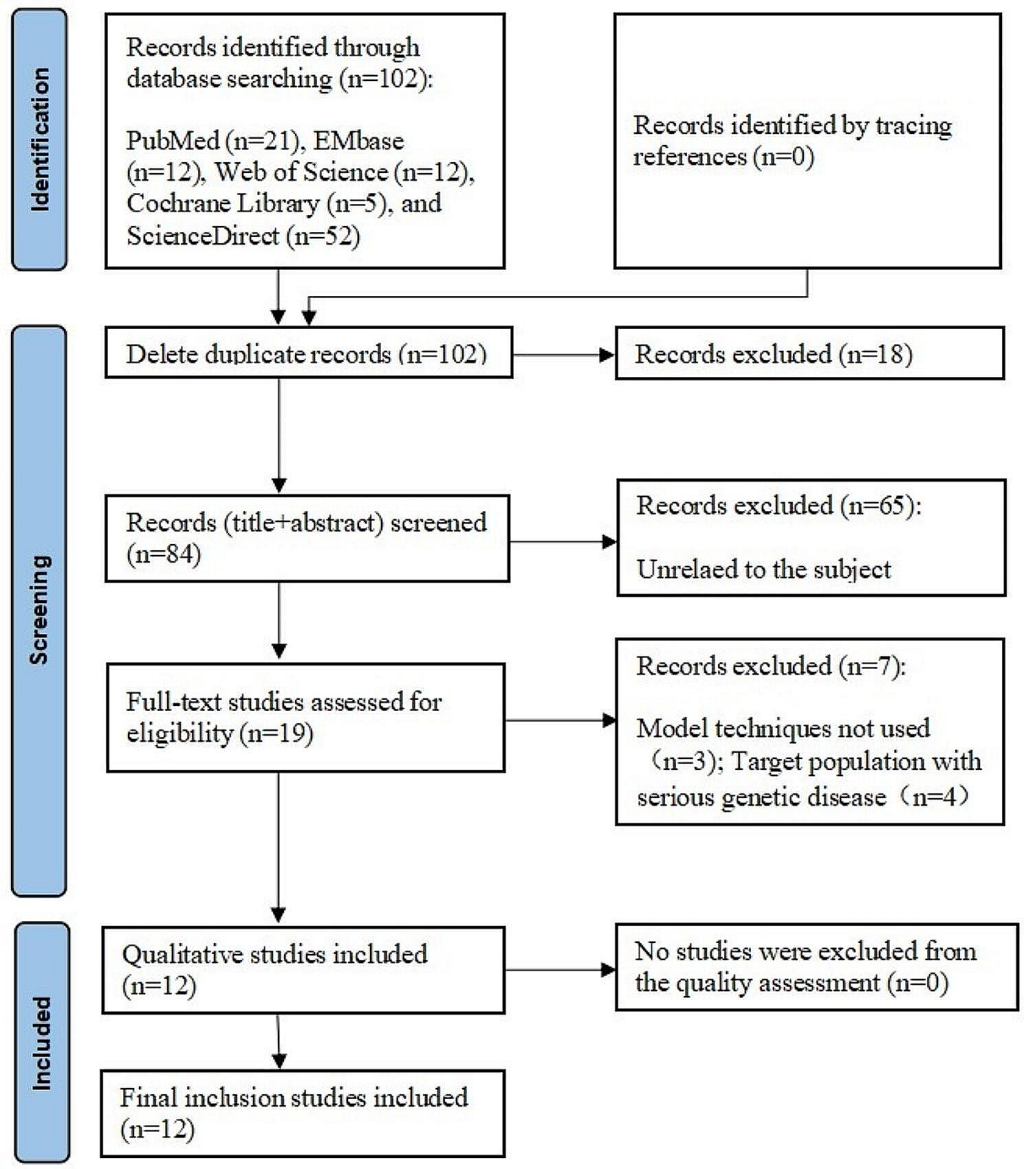
PRISMA flowchart

One hundred and two related articles were obtained in the initial examination, and 12 articles [13–24] were finally included after a layer-by-layer screening. The literature screening process and results are depicted in Fig. 2.

The quality evaluation results and basic characteristics of the included studies are presented in Tables 1 and 2, respectively. The reports of the 12 included studies were of high quality. Among them, three studies [13, 18, 23] did not explain the research perspective; only three studies [16, 18, 22] stated that the discount rate was not set; the model types were defined in all the 12 studies, but Mathieu (2020) and van Eekelen (2021) did not provide a specific model structure diagram. There were no subgroups, heterogeneity analysis, and distribution effect analysis in nine studies [13–16, 18–20, 22, 24], and there were no uncertainty analysis in two studies [18, 19].

**Table 1:** Quality evaluation results of economic evaluation of ART

Studies included	Items																												Results
	1	2	3	4	5	6	7	8	9	10	11	12	13	14	15	16	17	18	19	20	21	22	23	24	25	26	27	28	
Evans 2019 [13]	Y	Y	Y	Y	Y	Y	Y	*N*	Y	*N*	Y	Y	Y	Y	Y	Y	Y	*N*	*N*	Y	Y	Y	Y	Y	*N*	Y	Y	Y	23/28(82%)
Xue 2019 [14]	Y	Y	Y	Y	Y	Y	Y	Y	Y	*N*	Y	Y	Y	Y	Y	Y	Y	*N*	*N*	Y	Y	Y	Y	Y	Y	Y	Y	Y	25/28(89%)
Oostingh 2019 [15]	Y	Y	Y	Y	Y	Y	Y	Y	Y	*N*	Y	Y	Y	Y	Y	Y	Y	*N*	*N*	Y	Y	Y	Y	Y	*N*	Y	Y	Y	24/28(86%)
Jing 2020 [16]	Y	Y	Y	Y	Y	Y	Y	Y	Y	Y	Y	Y	Y	Y	Y	Y	Y	*N*	*N*	Y	Y	Y	Y	Y	Y	Y	Y	Y	26/28(93%)
Kluber 2020 [17]	Y	Y	Y	Y	Y	Y	Y	Y	Y	*N*	Y	Y	Y	Y	Y	Y	Y	Y	Y	Y	Y	Y	Y	Y	Y	Y	Y	Y	27/28(96%)
Mathieu 2020 [18]	Y	Y	Y	Y	Y	Y	Y	*N*	Y	Y	Y	Y	Y	Y	Y	Y	Y	*N*	*N*	*N*	Y	Y	Y	*N*	Y	Y	Y	Y	23/28(82%)
Sitler 2020 [19]	Y	Y	Y	Y	Y	Y	Y	Y	Y	*N*	Y	Y	Y	Y	Y	Y	Y	*N*	*N*	*N*	Y	Y	Y	*N*	*N*	Y	*N*	*N*	20/28(71%)
Facadio 2021 [20]	Y	Y	Y	Y	Y	Y	Y	Y	Y	*N*	Y	Y	Y	Y	Y	Y	Y	*N*	*N*	Y	Y	Y	Y	Y	*N*	Y	Y	Y	24/28(86%)
Lee 2021 [21]	Y	Y	Y	Y	Y	Y	Y	Y	Y	*N*	Y	Y	Y	Y	Y	Y	Y	Y	Y	Y	Y	Y	Y	Y	Y	Y	*N*	Y	26/28(93%)
van Eekelen 2021 [22]	Y	Y	Y	Y	Y	Y	Y	Y	Y	Y	Y	Y	Y	Y	Y	Y	Y	*N*	*N*	Y	Y	Y	Y	Y	Y	Y	Y	Y	26/28(93%)
Cheng 2021 [23]	Y	Y	Y	Y	Y	Y	Y	*N*	Y	*N*	Y	Y	Y	Y	Y	Y	Y	Y	Y	Y	Y	Y	Y	Y	*N*	Y	Y	*N*	24/28(86%)
Schwarze 2022 [24]	Y	Y	Y	Y	Y	Y	Y	Y	Y	*N*	Y	Y	Y	Y	Y	Y	Y	*N*	*N*	Y	Y	Y	Y	Y	Y	Y	Y	Y	25/28(89%)

Note: 1.Title; 2.Abstract; 3.Background and objectives; 4.Health economic analysis plan; 5.Study population; 6.Setting and location; 7.Comparators; 8.Perspective; 9.Time horizon; 10.Discount rate; 11.Selection of outcomes; 12.Measurement of outcomes; 13.Valuation of outcomes; 14.Measurement and valuation of resources and costs; 15.Currency, price date, and conversion; 16.Rationale and description of model; 17.Analytics and assumptions; 18.Characterizing heterogeneity; 19.Characterizing distributional effects; 20.Characterizing uncertainty; 21.Approach to engagement with patients and others affected by the study; 22.Study parameters; 23.Summary of main results; 24.Effect of uncertainty; 25.Effect of engagement with patients and others affected by the study; 26.Study fifindings, limitations, generalizability, and current knowledge; 27.Source of funding; 28.Conflflicts of interest

**Table 2:** Basic characteristics of the included studies

Studies included	Countries	Perspective	Study population	Comparators
Evans 2019 [13]	US	—	Patients receiving fresh/frozen embryo transfer	Different ovulation agents: ①Progesterone: ②Conventional GnRH
Xue 2019 [14]	Germany	Payer	Patients during an assisted reproductive cycle	Different ovulation agents: ①r-FSH: ②r-FSH biosimilars
Oostingh 2019 [15]	Netherlands	①Health system; ②The whole society	Low fertility couples undergoing IVF for the first time	Different pre-pregnancy care: ①mHealth coaching program; ②Usual care
Jing 2020 [16]	China	Patient	Patients aged 20 to 38 years undergoing their first IVF cycle	Different ovulation induction regiments: ①GnRH-a long-protocol; ②GnRH-A protocol
Kluber 2020 [17]	Germany	Patient	Women who plan to delay pregnancy until after the age of 40	Different interventions: ①oocyte cryopreservation; ②natural conception;③IVF/ICSI
Mathieu 2020 [18]	France	—	Women younger than 40 years of age diagnosed with endometriosis and referred for family planning	Different ovulation induction regiments: ① PPOS protocol; ②antagonist protocol
Sitler 2020 [19]	US	①Patient; ②Payer	Patients aged 20 to 44 undergoing IVF	Different transfer protocols: ① SET; ② DET
Facadio 2021 [20]	US	Payer	Patients undergoing IVF treatment with fresh donor oocytes	PGT-A
Lee 2021 [21]	US	①Patient; ②Payer	Women over 18 years of age undergoing their first fresh IVF/ frozen embryo transfer cycle	PGT-A
van Eekelen 2021 [22]	Netherlands	The whole society	Couples with unexplained low fertility	Different ovulation agents: ① CC; ②Letrozole; ③Gonadotrophins
Cheng 2021 [23]	US	—	Couples who have undergone vasectomy reversal (couples with advanced maternal age)	Different treatment options: ①VR followed by NC; ②SR with IVF; ③VR and SR followed by failed NC and then IVF; ④VR and SR followed by failed IVF and then NC
Schwarze 2022 [24]	Spain	Health system	Fresh embryo transfer in patients with ART cycles	Different ovulation agents: ①Originator r-hFSHα; ②r-hFSHα biosimilars

Note: — represents what is not explained in the text (the same below)

CC: clomiphene citrate, DET: double embryo transfer, FSH: follicle stimulating hormone, GnRH: gonadotropin-releasing hormone, GnRH-a: gonadotropin-releasing hormone-agonist, GnRH-A: gonadotropin-releasing hormone-antagonist, ICSI: intracytoplasmic sperm injection, IVF: in vitro fertilization, NC: natural conception, PGT-A: preimplantation genetic testing for aneuploidy, PPOS: progestin-primed ovarian stimulation, r-FSH: recombinant follicle stimulating hormone, r-hFSHα: recombinant human follicle stimulating hormone alfa, SET: single embryo transfer, SR: sperm retrieval, US: united states, VR: vasectomy reversal

### Sources of studies

Five studies [13, 19–21, 23] were conducted in the United States (US), which is the country with the highest number of studies, followed by two studies each on Germany [14, 17] and the Netherlands [15, 22]. The remaining three studies were conducted in China [16], France [18], and Spain [24] (Table 2). As a pioneer of ART, most of the technologies originated in the US. IVF technology started in the US [25, 26], so it has the most research. In addition, the Society For ART (SART) [27] was established in the US. More than 90% of the assisted reproductive institutions in the US were registered and certified by SART, and the success of ART in the US is largely due to SART [28]. With the rapid development of ART in some countries, other countries also began to carry out relevant research on it.

### Population

All studies were conducted on low-fertility couples, and other characteristics of the population varied among the studies, and the choice of the target population was also related to the specific purpose of the study. Six studies [16–19, 21, 23] restricted the age of patients. Further, six studies [13, 15, 16, 20, 21, 24] limited the duration of treatment (the first IVF cycle) and treatment options (fresh/frozen embryo transfer). The patients in one study [18] had additional complications (endometriosis). Two studies [14, 22] did not place additional restrictions on patients (Table 2).

### Comparative scheme

The studies evaluated different ovulation agents, different ovulation induction regiments, different transplantation regiments, and other different interventions. Four studies [13, 14, 22, 24] evaluated different ovulation agents: progesterone, GnRH and its analogs, r-FSH and its biosimilars, gonadotropins, as well as Letrozole and clomiphene. Two studies [16, 18] evaluated different ovulation induction regiments—agonist length regiments, antagonist regiments, and PPOS regiments. One study [19] evaluated different transfer protocols—single or double embryo transfer. Two studies [20, 21] evaluated the economics of genetic testing before embryo transfer. Three studies evaluated different pre-pregnancy care [15], whether oocytes were cryopreserved [17], and different treatment options [23]. (Table 2)

### Research perspectives

Different research perspectives have different ranges of inclusion costs. The China Pharmacoeconomic Evaluation Guide (2020 edition) [29] recommends the whole society and health system perspectives for evaluation. However, researchers can choose the appropriate research perspective based on the research purpose. For example, if the research results are to help with the selection of a national medical insurance drug catalog, the perspective of the medical insurance payer should be selected. The research perspectives of the studies include patients’ perspective, medical insurance payers’ perspective, health systems’ perspective, and the whole society’s perspective. Among them, two studies [16, 17] conducted the evaluation from the perspective of patients. Two studies [14, 20] evaluated from the perspective of medical insurance payers. One study [24] evaluated from the perspective of health systems. One study [22] evaluated from the perspective of the whole society. One study [15] evaluated from the perspectives of both the health system and the whole society. Two studies [19, 21] evaluated from the perspectives of patients and medical insurance payers (Table 2).

### Model type

At present, there are five commonly used economic evaluation models—the decision tree model, Markov model, partition survival model, discrete event simulation model, and system dynamics model [29, 30]. The models employed in the 12 studies are decision tree, Markov, decision tree plus Markov, and multiple regression models. Among them, nine studies [13–16, 19–22, 24] adopted decision tree, accounting for the largest proportion. One study [17] adopted Markov; one study [23] adopted the decision tree plus Markov, and one study [18] adopted the multiple regression model (Table 3). The decision tree model is suitable for the pharmacoeconomic evaluation of transient diseases with a short research time and clear treatment results, as it is a static short-term simulation [29]. Markov, the partition survival model, and the discrete event simulation model are dynamic models that can be simulated for a long time and are suitable for the evaluation of chronic diseases that change with time [29]. So far, no study has been conducted on the pharmacoeconomic evaluation of ART with the partition survival model and the discrete event simulation model.

**Table 3:** Model features of the included studies

Studies included	Model type	Healthy state	Cycle	Time horizon	Discount rate	WTP
Evans 2019 [13]	Decision tree	①Live birth; ②No live birth	The first assisted reproductive cycle resulted in a live birth	An assisted reproductive cycle, Two embryo transfer	—	—
Xue 2019 [14]	Decision tree	①Oocyte retrieval; ②No oocyte retrieval; ③Embryo transfer: ④No embryo transfer: ⑤Pregnancy: ⑥No pregnancy: ⑦Live birth: ⑧Miscarriage	An assisted reproductive cycle	An assisted reproductive cycle	—	—
Oostingh 2019 [15]	Decision tree	①Pregnant; ②Not pregnant	One IVF cycle (24 weeks)	Two IVF cycles	—	—
Jing 2020 [16]	Decision tree	①Oocytes; ②No oocytes; ③Embryos; ④No embryos; ⑤Ongoing pregnancy; ⑥No ongoing pregnancy	One IVF cycle (up to ongoing pregnancy)	Fresh embryo transfer: one IVF cycle; Frozen embryo transfer: until all available embryos are used up or pregnancy continues	No consider	—
Kluber 2020 [17]	Markov	①Live birth; ②No live birth	1 year	5 years	—	—
Mathieu 2020 [18]	Multiple regression model	Not	One cycle of ovarian stimulation	One cycle of ovarian stimulation	No consider	—
Sitler 2020 [19]	Decision tree	①Ongoing pregnancy; ②No ongoing pregnancy; ③Singleton; ④Twin	One IVF cycle (until live birth)	Single embryo transfer model: two IVF cycles; Double embryo transfer model: one IVF cycle	—	—
Facadio 2021 [20]	Decision tree	①Development of a blastocyst embryo; ②PGT-A testing; ③Blasto cyst transfer; ④Clinical pregnancy; ⑤Miscarriage; ⑥Pregnancy termination: ⑦Live birth	One IVF cycle (until live birth)	One IVF cycle	—	$50,000 per additional live birth
Lee 2021 [21]	Decision tree	①No eggs; ②Egg retrieva; ③Cycle cancelled before ER; ④Embryo transfer; ⑤No embryo transfer; ⑥No pregnancy; ⑦Miscarriage; ⑧Singleton live birth: ⑨Twin live birth: ⑩HOM live birth	One IVF cycle (up to live birth)	Until live birth or 12 months after ovarian stimulation	—	Not set
van Eekelen 2021 [22]	Decision tree	—	One IUI-OS cycle	Four IUI-OS cycles (within one year)	No consider	A live birth value: ①€1-€3000; ②€3000-€55,000; ③€55,000 OR more
Cheng 2021 [23]	Decision tree + Markov	①Natural conception success; ②Natural conception failure; ③IVF/ICSI success; ④IVF/ICSI failure	One natural conception attempt or one IVF/ICSI attempt	One year after intervention	—	—
Schwarze 2022 [24]	Decision tree	①Pregnancy; ②No pregnancy; ③ Live birth; ④ No live birth	One cycle of ART with fresh embryo transfer (until live birth)	One treatment cycle	—	€20,000 per live birth (cost-effectiveness acceptable curve)

ER: egg retrieval, HOM: higher-order multiple, ICSI: intracytoplasmic sperm injection, IUI-OS: intrauterine insemination-ovarian stimulation, IVF: in vitro fertilization, PGT-A: preimplantation genetic testing for aneuploidy

### Health states

There were many health states of the models included in the study, mainly including with/without oocytes, (not) to take the eggs, with/without embryo, (not) embryo transfer, (not) pregnant, (not) ongoing pregnancy, abortion, (not) live birth, naturally conceived success/failure, IVF/ICSI success/failure, cancel the cycle, and single/multiple births (Table 3). Assisted reproduction has a long cycle; many links are involved [31], and many health states are included. Based on the differences in research population, research purpose, and research design, the health states included in the models constructed by different studies are different, and the model structure is not unified.

### Cycle period and time horizon

Regarding the cycle period and time horizon, the cycle of nine studies [13–16, 19–21, 23, 24] was a complete assisted reproduction treatment cycle (until ongoing pregnancy or live birth). Based on the model assumptions, the time horizons were different, including an assisted reproductive cycle [13, 14, 16, 19, 20, 24], two auxiliary reproductive cycles [15, 19], a year after the intervention [21, 23], or usable embryos is all used up [16]. Two studies [18, 22] had one cycle of ovarian stimulation, and the time horizon was one ovarian stimulation cycle [18] and four ovarian stimulation cycles (within 1 year) [22]. There was also a one-year study [17] with a five-year time horizon (Table 3). Generally, an assisted reproductive cycle is three months. The cycle of these studies is set based on the assisted reproductive treatment cycle, and the time horizon is generally short, which is related to the particularity of the assisted reproductive treatment (e.g., long treatment cycle, high withdrawal rate, age, and other factors) [8, 32].

### Discount rate

Of the 12 studies included, only three [16, 18, 22] explained why no discount rate was set (Table 3). These three studies pointed out that the duration of ART economics evaluation is usually from the start of treatment to achieving (ongoing) pregnancy or live birth, with a short duration and relatively stable cost. Moreover, it usually does not consider the discount rate. The duration of the economic evaluation for some chronic diseases [12, 33, 34] is usually long (50 years or even throughout a lifetime), so it is necessary to set a discount rate.

### Willingness to pay (WTP)

In economic evaluation, when a strategy is more effective and less costly, it is an absolute dominant strategy. When a strategy is less effective and more costly, it is an absolutely dominated strategy. When the effectiveness and cost of a strategy are higher, a WTP helps to determine whether the strategy is cost-effective [21]. The WTP range recommended by the China Pharmacoeconomic Evaluation Guide (2020 edition) [29] is 1–3 times the per capita GDP of a country, but this range is not applicable in assisted reproduction. Out of the 12 studies included, eight studies [13–19, 23] did not specify the WTP. One US study [20] set the WTP at $50,000 per extra live birth. One Dutch study [22] assumed that each live birth had a certain monetary value, and the net monetary benefit (NMB) value of each protocol could be obtained. The results were represented by the NMB curve, and the results showed that the most economical protocols under different ranges of monetary value per live birth were different. One Spanish study [24] set the WTP at €20,000 per live birth through a cost-effectiveness acceptability curve. There was also one US study [21] that did not set a discount rate (Table 3) because the WTP usually refers to the value of one year of life or quality-adjusted life years—a range that is difficult to translate into birth outcomes. In a multicenter randomized controlled trial, van Hoogenhuijze (2022) [35] also pointed out that there is still an evidence gap in the current WTP guidelines for fertility as the acceptability of expenditure for an additional live birth cannot be clearly defined.

### Model parameters

For the same economic evaluation, different sources of model parameters lead to different results. In the studies included in the analysis, the direct medical cost data mainly came from published literature, clinical data of the included patients (retrospective/prospective), medical institution data, and other public data. Most of the studies were not limited to a single source but combined data from multiple sources. Direct non-medical costs mostly refer to transportation costs, which are the average ticket price from each city to the target city. Indirect costs, also known as labor costs, refer to the loss of labor time and productivity of patients and their families caused by illness, disability or death. This includes the loss of wages of patients and their families caused by suspension of school, rest from work, and early death, and consist of lost working days and per capita disposable income. Regarding the effect data, four studies [16, 18, 19, 21] obtained them from the clinical data of the included patients, among which only one study [18] was based on a prospective database. Six studies [13, 14, 20, 22–24] obtained their data from published literature. The other two obtained their data from a randomized controlled trial [15] and a German IVF registry [17]. The data on state transition probability were mainly derived from published literature, clinical data of the included patients, and publicly available national data. Eight studies [13–15, 17, 20, 22–24] referred to published literature. Three studies [16, 19, 21] obtained their data from the clinical data of the included patients. Three studies [13, 17, 23] referred to the national public data, among which the data of two US studies [13, 23] were from SART, and the data of one German study [17] was from the German IVF registry. (Table 4)

**Table 4:** Model parameters of the included studies

Studies included	Sources of cost data	Sources of effect data	Sources of state transition probability data	Cost inclusion item
Evans 2019 [13]	①Published literature; ②Medical websites	Published literature	①Published literature; ②SART data	Direct medical costs: ①IVF; ②Embryo transfer; ③Drugs
Xue 2019 [14]	①Published literature; ②Publicly available German information	Published literature	Published literature	Direct medical costs: ① Assisted reproduction and birth costs; ② Adverse event costs; ③Drug costs
Oostingh 2019 [15]	Published literature	Randomized controlled trial	Published literature	Health systems’ perspective Direct medical costs: ①Costs related to the mHealth program; ②IVF; ③Other relevant health care costs The whole society’s perspective Cost of inclusion from a health systems’ perspective + Costs outside the health care sector (e.g., costs due to absence at work)
Jing 2020 [16]	①Clinical data of infertility patients were retrospectively included; ②Direct non-medical costs: the average fare from cities in Zhejiang province to Hangzhou: ③Indirect cost: per capita disposable income	Clinical data of infertility patients were retrospectively included	Clinical data of infertility patients were retrospectively included	Direct medical costs: ①Drugs; ②Ultrasound: ③Laboratory: ④Surgery (Including the costs of treatment for OHSS): ⑤Care Direct non-medical costs: Transportation Indirect costs: Cost of lost work
Kluber 2020 [17]	①Current average price range in Germany; ②German medical fee schedule: ③German IVF Registry	German IVF Registry	①German IVF Registry; ②Published literature	Direct medical costs: ①Ovarian stimulation medication: ②Oocyte retrieval: ③Oocyte freezing: ④Embryo transfer: ⑤Oocyte storage
Mathieu 2020 [18]	①Clinical data of included patients (prospective database): ②State fixed price	Clinical data of included patients (prospective database)	Not	All direct medical costs associated with family planning procedures
Sitler 2020 [19]	①Clinical data of the patients were retrospectively included: ②U.S. Army Women’s Product Line	Clinical data of the patients were retrospectively included	①Clinical data of the patients were retrospectively included: ②ICD-10 diagnostic code	Direct medical costs: ①Progesterone: ②Ultrasound visits: ③Laboratory: ④ICSI: ⑤Embryo transfer: ⑥Delivery: ⑦Maternal and neonatal costs
Facadio 2021 [20]	①Published literature: ②Author agency internal data	Published literature	Published literature	Direct medical costs: ①IVF: ②Donor eggs/sperm: ③Embryo transfer: ④PGT-A: ⑤Miscarriage: ⑥Prenatal care: ⑦Amniocentesis: ⑧ Genetic counseling: ⑨Live birth
Lee 2021 [21]	①Published literature: ②Retrospective inclusion of patient clinical data reported to the SART CORS	Retrospective inclusion of patient clinical data reported to the SART CORS	Retrospective inclusion of patient clinical data reported to the SART CORS	Patients’ perspective Direct medical costs: All costs associated with IVF Payers’ perspective Direct medical costs: The aforementioned IVF costs and obstetrical costs related to prenatal care, miscarriage management, and birth
van Eekelen 2021 [22]	①Dutch Formulary on Medication: ②An expert panel on cost-effectiveness from the Dutch consortium for Research in Women’s Health (Data were collected from two university hospitals and one general hospital)	Published literature + formulas	Published literature	Direct medical costs: ①Drugs: ②Delivery of a multiple pregnancy
Cheng 2021 [23]	Two high capacity academic centres	Published literature	①Published literature: ②SART Annual Report	Direct medical costs: Out-of-pocket expenses for patients
Schwarze 2022 [24]	①Published literature: ②Clinical expert validation: ③Publicly available Spanish information (Spanish Ministry of Health, etc.)	Published literature	Published literature	Direct medical costs: ①Treatment: ②Medication: ③Pregnancy follow-up: ④Live birth: ⑤Miscarriage: ⑥Severe OHSS

ICSI: intracytoplasmic sperm injection, IVF: in vitro fertilization, OHSS: ovarian hyperstimulation syndrome, PGT-A: preimplantation genetic testing for aneuploidy, SART: Society for ART, SART CORS: Society for ART Clinical Outcome Reporting Systems

In the economic evaluation of ART, direct medical costs mainly include hospital costs such as drug, egg collection, embryo transfer, ultrasound, laboratory, delivery, abortion, and adverse event treatment costs. Only three [14, 16, 24] of these studies explicitly included the cost of treating adverse events. Direct non-medical costs include medical transportation, accommodation, and nursing costs. Indirect costs refer to patient-related labor loss, income loss caused by accompanying family members for medical treatment, etc. One study [15] included indirect costs, and another study [16] included direct non-medical and indirect costs, where direct non-medical costs included only transportation costs. (Table 4)

### Results of the studies

ART quality control indicators include the number of eggs harvested, clinical pregnancy rate, implantation rate, early abortion rate, ovarian hyperstimulation syndrome (OHSS) incidence, multiple pregnancy rate, delivery rate, live birth rate, and cumulative live birth rate [36]. Six studies [13, 14, 17, 20, 21, 24] used live birth rate as a common outcome measure. The outcome measures of the remaining six studies were the number of ongoing pregnancies [15], ongoing pregnancy rate and cumulative ongoing pregnancy rate [16], number of eggs harvested [18], number of delivery [19], cumulative live birth rate [22], and quality-adjusted life years [23]. Cheng et al. used QALYs as an outcome indicator, and these values were obtained from a study by Scotland [37], which evaluated the cost-utility of single and double embryo transfers. At present, the live birth rate is the most used indicator to measure the effect of ART. The indicators used in cost–benefit analyses are the cost-effectiveness ratio and incremental cost-effectiveness ratio. Except that in one study [18], the clinical effect of the two schemes was the same, and the least cost analysis method was used. Sensitivity analysis was carried out to test the robustness of the model. Sensitivity analysis was not carried out in two studies [18, 19], and the sensitivity analysis methods for the remaining 10 studies included one-way sensitivity analysis, deterministic sensitivity analysis, probabilistic sensitivity analysis, and scenario analysis. When analyzing the research results, different model assumptions, different outcome indicators considered, and different WTP will affect the conclusion (Table 5).

**Table 5:** Results of the included studies

Studies included	Outcome indicators	Cost benefit analysis	Sensitivity analysis	Study findings
Evans 2019 [13]	Live-birth rate	Cost effectiveness ratio	①Deterministic sensitivity analysis: ②Scenario analysis	Progestin protocols were not cost-effective compared with conventional fresh embryo transfer protocols. Progestin use, however, may be cost-effective when freeze-only is planned such as in preimplantation genetic testing or fertility-preservation cycles where a GnRH antagonist protocol would otherwise be used.
Xue 2019 [14]	Live-birth rate	①Cost effectiveness ratio: ②Incremental cost-effectiveness ratio	①One-way sensitivity analysis: ②Scenario analysis	Treatment with the Originator could result in a lower cost per live birth in comparison to biosimilars.
Oostingh 2019 [15]	The number of ongoing pregnancies	Incremental cost-effectiveness ratio	①Probabilistic sensitivity analysis: ②Deterministic sensitivity analysis	The mHealth coaching program Smarter Pregnancy is potentially cost saving for subfertile couples preceding their first IVF treatment.
Jing 2020 [16]	①Ongoing pregnancy rate: ②Cumulative ongoing pregnancy rate	①Cost effectiveness ratio: ②Incremental cost-effectiveness ratio	Deterministic sensitivity analysis	In fresh embryo transplantation cycle, the GnRH-antagonist protocol has economic advantage. However, the GnRH-agonist long protocol is more cost efective considering the cumulative ongoing pregnancy rate in the fresh embryo and frozen embryo transplantation cycles.
Kluber 2020 [17]	Live-birth rate	Incremental cost-effectiveness ratio	①Scenario analysis: ②One-way sensitivity analysis: ③Probabilistic sensitivity analysis: ④Deterministic sensitivity analysis	Social freezing in Germany lead to additional pregnancies among women over 40 but also to signifcantly higher costs, since given the live birth success rates and pricing, social freezing does not appear to be cost-efective.
Mathieu 2020 [18]	The number of eggs harvested during the cycle	The least cost analysis (Same clinical effect)	without	Antagonist and PPOS protocols were associated with similar results but the medico-economic analysis was in favor of PPOS protocols.
Sitler 2020 [19]	Number of delivery	Cost effectiveness ratio	without	SET in a system with no infertility coverage saves approximately $3.5 million per 250 patients. Higher personal costs as seen with SET may incentivize patients to seek DET.
Facadio 2021 [20]	Live-birth rate	Incremental cost-effectiveness ratio	①One-way sensitivity analysis: ②Probabilistic sensitivity analysis	PGT-A is not cost-effective to achieve live births in patients using fresh donor oocytes.
Lee 2021 [21]	Live-birth rate	The primary outcome: Incremental costs of the two treatment strategies; Secondary outcome: Incremental cost-effectiveness ratio	One-way sensitivity analysis	From an economic perspective, routine preimplantation genetic testing for aneuploidy should not be universally adopted; however, it may be costeffective in certain scenarios.
van Eekelen 2021 [22]	Cumulative live birth rate	Incremental cost-effectiveness ratio	Scenario analysis	In settings where a live birth is valued at €3000 or less, between €3000 and €55,000 and above €55 000, clomiphene citrate (CC), Letrozole and gonadotrophins were the most cost-effective option in terms of net benefit, respectively.
Cheng 2021 [23]	Quality-adjusted life years	Cost effectiveness ratio	Deterministic sensitivity analysis	In couples with a history of vasectomy and female of AMA, it is most cost-effective to undergo a VR. If the couple opts for SR for IVF, it is more cost-effective to undergo a concomitant VR than SR alone.
Schwarze 2022 [24]	Live-birth rate	①Cost effectiveness ratio: ②Incremental cost-effectiveness ratio	①One-way sensitivity analysis: ②Probabilistic sensitivity analysis	The results indicate that originator r-hFSH-alfa has a 100% probability of being cost effective considering a WTP threshold of V20,000 versus r-hFSH-alfa biosimilars for OS prior to ART treatment in fresh embryo transfer ART cycles.

### Subgroup analysis

Among the 12 studies included in this study, only Kluber et al. [17], Lee et al. [21] and Cheng et al. [23] set subgroups. These three studies all carried out subgroup analysis on women of different ages, and finally showed that different ages of women would have an impact on the research results.

## Discussion

This study adopts a qualitative analysis method to systematically evaluate domestic and foreign studies on ART-related economic evaluation and finds that there are differences and deficiencies in the report format, model framework, model parameters, and other aspects of these studies.

### Report format

In terms of the report format, the research population, research perspective, time horizon, model hypothesis, discount rate, parameter source, and other contents should be standardized in the report of the pharmacoeconomic evaluation research [11]. Previous studies mostly referred to the CHEERS 2013. CHEERS 2022 version has been released and has now become the new standard guidelines for preparing health economic assessment reports [38]. Thus, later economic evaluation should strictly adhere to this latest standard, list the reference guide, describe the included projects in full detail, and ensure transparency in the scientific process. The CHEERS 2022 version improves the quality and readability of reports.

### Model framework

In terms of model types, most of the studies included in the analysis used the decision tree model, whereas a few used the Markov model. The two models have their advantages and disadvantages. The Markov model can transform between multiple states and can be simulated for a long time [29], whereas the assisted reproductive treatment cycle is long. It is suggested to try to use the Markov model to evaluate ART economics in the future.

In terms of health status, there are many health statuses of models in the economic evaluation of ART at present. The health statuses of models constructed by various studies are very different, and the model structure is not unified. In the future, further discussion is needed to form a mature and unified model structure to promote the further development of ART economic evaluation research.

### WTP

In terms of WTP, the WTP range recommended by the China Pharmacoeconomic Evaluation Guide (2020 edition) [29] is not applicable to assisted reproduction. Although some countries have conducted relevant studies on the WTP range of ART, these studies [39–42] revealed that the WTP value is highly uncertain, and there are still gaps in the WTP guidelines for fertility. Therefore, few studies set the WTP when conducting ART economic evaluation. It is suggested that when the threshold is uncertain, it can be assumed that each live birth has a certain monetary value [22]. Then the cost and benefit of the intervention are monetized and can be directly compared, which is a more appropriate method to evaluate the economics of infertility treatment [43], and can compensate for the uncertainty caused by the lack of threshold to a certain extent. And the determination of WTP threshold of ART is the place that experts need to pay attention to in the future.

### Model parameters

In terms of the source of model parameters, at present, the model parameters of relevant studies are mainly derived from published literature and clinical data of included patients (retrospective), only a few studies have used prospective data. Most studies have narrow sources of data and lack real-world evidence (data from long-term follow-up of patients). The construction of the model requires a large number of assumptions, the scenario is idealistic and different from clinical practice, which leads to potential bias in the results of studies and may not be applicable to other institutions or countries, with insufficient universality. The US ART achievements are largely due to SART [27], which is the largest association of assisted reproduction. China lacks such large, publicly available data sources to support the related research, and few prospective randomized controlled trials have been conducted. Future studies should use more large samples, multicenter prospective randomized controlled trials [16], and long-term follow-up of patients so that the research results would be more credible and universal.

In terms of cost inclusion, most studies only included direct medical costs in the analysis, whereas few included direct non-medical and indirect costs, which is not enough to truly reflect the treatment situation of the infertility population, leading to deviation of results. For cost estimates, we suggest that direct non-medical and indirect costs should be included. Moreover, the analysis should be conducted from the perspective of the whole society, as it is the most comprehensive research perspective, including the perspectives of all stakeholders in the healthcare field. This perspective reflects the various social opportunity costs associated with different interventions [44]; this will make the research results more comprehensive and credible.

In terms of the outcome indicators, related studies generally take live birth rate as the clinical outcome indicator of ART, only a few studies have considered the implantation rate, clinical pregnancy rate, delivery rate, etc. Moreover, the influence of adverse events (mainly OHSS) on clinical outcomes has been considered by a few studies. The indicators of health outcomes are mostly clinical effects, which cannot assess the satisfaction of patients during treatment. Therefore, the evaluation index of the clinical outcome of ART is relatively single. When related studies are conducted in the future, the therapeutic effect can be comprehensively evaluated by combining the efficacy and safety indicators, so as to make the evaluation results more comprehensive and credible.

### Subgroup analysis

Economic evaluation is usually performed at the level of the overall target population, but can also be performed at the level of subgroups as needed. Subgroup analysis can be conducted according to population characteristics, disease subtypes, severity, and comorbidities to resolve the uncertainty and robustness of research conclusions. The economic benefits of the same intervention for different subgroups of people may be different. In ART, female age is a key factor affecting the success rate of fertility treatment, and some studies have shown that the probability of pregnancy decreases with the increase of female age [45]. Among the studies included in this systematic review, subgroup analysis was carried out in three studies [17, 21, 23] based on different age groups of women. Therefore, it is suggested that subgroup analysis based on age stratification should at least be considered in future economic evaluation.

## Limitations

There are some shortcomings in this system evaluation. (1) This system evaluation only includes English articles, which may lead to an incomplete analysis. (2) CHEERS 2022 was the reference standard for the literature quality evaluation. The included studies may have referred to other standards when writing their reports, which may have resulted in inappropriate literature quality evaluation. (3) As there are few economic evaluations of ART in China, out of the 12 studies, only one study [16] included in the analysis is domestic, and the rest are all foreign studies. It is hoped that more relevant studies would be carried out in China, and systematic evaluation would be conducted for domestic studies to put forward more targeted and suitable opinions and suggestions for China.

## Conclusion

To sum up, the overall quality of the studies included in the system evaluation is high, but there are still differences and deficiencies in the report format, model framework, and other aspects. We suggest that the reports should be written in strict accordance with the standard guidelines on the economic evaluation of ART. The setting of the threshold range in the field of fertility should be actively discussed, and the monetary value of each live birth is assumed to be in a certain range when the WTP threshold for fertility is uncertain. The range of the parameter sources should be expanded. Direct non-medical and indirect costs should be included in the calculation of costs, and the analysis should be carried out from the perspective of the whole society. In the evaluation of clinical effect, the effectiveness and safety indexes should be selected for a comprehensive evaluation, thereby making the evaluation more comprehensive and reliable. At least subgroup analysis based on age stratification should be considered in the relevant economic evaluation.

## Data Availability

All data analyzed as part of this study are included in this article.

## Abbreviations

ART: Assisted Reproductive Technology
CC: Clomiphene Citrate
CHEERS: Comprehensive Health Economics Evaluation Report Standard
DET: Double Embryo Transfer
ER: Egg Retrieval
ET: Embryo Transfer
FSH: Follicle Stimulating Hormone
GIFT: Gamete Intrafallopian Transfer
GnRH-a: Gonadotropin-Releasing Hormone-agonist
GnRH-A: Gonadotropin-Releasing Hormone-Antagonist
GnRH: Gonadotropin-Releasing Hormone
HOM: Higher-Order Multiple
ICMART: International Committee Monitoring Assisted Reproductive Technology
ICSI: Intracytoplasmic Sperm Injection
IUI-OS: Intrauterine Insemination-Ovarian Stimulation
IVF: In Vitro Fertilization
NC: Natural Conception
OHSS: Ovarian Hyperstimulation Syndrome
PGT-A: Preimplantation Genetic Testing for Aneuploidy
PGT: Preimplantation Genetic Testing
PPOS: Progestin-Primed Ovarian Stimulation
r-FSH: Recombinant Follicle Stimulating Hormone
r-hFSHα: Recombinant Human Follicle Stimulating Hormone alfa
SART: Society for Assisted Reproductive Technology
SET: Single Embryo Transfer
SR: Sperm Retrieval
UNFPA: United Nations Population Fund
US: United States
VR: Vasectomy Reversal
WHO: World Health Organization
WTP: Willingness To Pay
